# Awareness of Infectious Disease Screening During Early Pregnancy and Knowledge About its Vertical Transmission in Japan: A Report from the Pregnant Women Health Initiative

**DOI:** 10.1007/s10995-023-03597-5

**Published:** 2023-02-08

**Authors:** Mizuha Odagami, Akiko Iwata, Kazumi Kubota, Kentaro Kurasawa, Mika Okuda, Shigeru Aoki, Tomoo Hirabuki, Tomoko Tujie, Haruya Saji, Tetsuya Hasegawa, Natsuko Kobayashi, Yutaka Ueda, Shinichi Ishioka, Takayuki Enomoto, Makoto Tsuji, Hiroyuki Shigeta, Kumi Koike, Hiroaki Tanaka, Rie Tsukinaga, Yoshimi Hasegawa, Reiko Numazaki, Hajime Ota, Hiroaki Kase, Hiroshi Ishikawa, Yoshihiro Saito, Takaharu Yamawaki, Etsuko Miyagi

**Affiliations:** 1 Department of Obstetrics and Gynecology, Yokohama City University Graduate School of Medicine, 3-9, Fukuura, Kanazawa-Ku, Yokohama City, Kanagawa 236-0004 Japan; 2 Department of Biostatistics, Yokohama City University, Yokohama, Japan; 3 Department of Obstetrics and Gynecology, National Hospital Organization Yokohama Medical Center, Yokohama, Japan; 4 Perinatal Center for Maternity and Neonates, Yokohama City University Medical Center, Yokohama, Japan; 5 Department of Obstetrics and Gynecology, Odawara Municipal Hospital, Odawara, Japan; 6 Department of Obstetrics and Gynecology, Toyonaka Municipal Hospital, Toyonaka, Japan; 7 Department of Obstetrics and Gynecology, Fujisawa Municipal Hospital, Fujisawa, Japan; 8 Department of Obstetrics and Gynecology, Yamato Municipal Hospital, Yamato, Japan; 9 Department of Obstetrics and Gynecology, Osaka University Hospital, Osaka, Japan; 10 Division of Perinatal Medicine, Sapporo Medical University Hospital, Sapporo, Japan; 11 Department of Obstetrics and Gynecology, Niigata University Medical and Dental Hospital, Niigata, Japan; 12 Department of Obstetrics and Gynecology, Saiseikai Matsuzaka General Hospital, Matsuzaka, Japan; 13 Department of Obstetrics and Gynecology, Yokohama Municipal Hospital, Yokohama, Japan; 14 Department of Obstetrics and Gynecology, Sado General Hospital, Sado, Japan; 15 Department of Obstetrics and Gynecology, Mie University Hospital, Tsu, Japan; 16 Department of Obstetrics and Gynecology, Yokosuka Kyosai Hospital, Yokosuka, Japan; 17 Department of Obstetrics and Gynecology, Saiseikai Yokohama-Shi Nanbu Hospital, Yokohama, Japan; 18 Department of Obstetrics and Gynecology, Yokohama Minami Kyosai Hospital, Yokohama, Japan; 19 Department of Obstetrics and Gynecology, Teine Keijinkai Hospital, Sapporo, Japan; 20 Department of Obstetrics and Gynecology, Nagaoka Chuo General Hospital, Nagaoka, Japan; 21 Department of Obstetrics and Gynecology, Kanagawa Children’s Medical Center, Yokohama, Japan; 22 Department of Obstetrics, Hokkaido University Hospital, Sapporo, Japan; 23 Department of Obstetrics and Gynecology, Japanese Red Cross Ise Hospital, Ise, Japan

**Keywords:** Infectious disease screening, Questionnaire survey, Mother-to-child transmission, Pregnant women

## Abstract

**Objectives:**

We aimed to clarify the accuracy of pregnant women’s knowledge and understanding regarding infectious disease screening in early pregnancy and clarify the roles that should be played by health care providers in promoting the health of pregnant women and their children.

**Methods:**

A cross-sectional questionnaire survey was conducted in 25 hospitals across Japan from May 2018 to September 2019. We compared the agreement rates regarding screening results for hepatitis B virus (HBV), hepatitis C virus (HCV), syphilis, human T-cell leukemia virus-1 (HTLV-1), and cervical cytology in the medical records and understanding of their results by pregnant women. We then investigated whether participants had knowledge regarding the risk of mother-to child transmission in these diseases and factors associated with their knowledge.

**Results:**

We enrolled 2,838 respondents in this study. The rates of agreement for HBV and cervical cancer screening related to human papillomavirus infection were “substantial,” those for syphilis was “moderate,” and those for HCV and HTLV-1 were “fair,” according to the Kappa coefficient. The rate of knowledge regarding mother-to-child transmission of syphilis was highest (37.0%); this rate for the other items was approximately 30%. Increased knowledge was associated with higher educational level and higher annual income.

**Conclusions for Practice:**

Pregnant women in Japan had generally good levels of understanding regarding their results in early-pregnancy infectious disease screening. However, they had insufficient knowledge regarding mother-to-child transmission of these diseases. Health care providers should raise awareness in infectious disease prevention among pregnant women and the general public, providing appropriate measures and implementing effective perinatal checkups and follow-ups for infectious diseases.

## Significance

A previous study reported biases in knowledge about mother-to-child disease transmission among pregnant women. However, there are no reports on the accuracy of pregnant women’s understanding and their backgrounds related to knowledge about infectious diseases. In this report, Japanese pregnant women had generally good understanding of their results in infectious disease screening. However, they had insufficient knowledge regarding mother-to-child transmission of infectious diseases. It is crucial for health care providers to raise awareness in infectious disease prevention and provide appropriate measures for pregnant women and the general public, such as implementing effective perinatal checkups and follow-ups.

## Objectives

In Japan, where the birth rate is decreasing and women are becoming pregnant at older ages, prenatal examinations for pregnant women are provided at public expense as a health examination. Local revenue measures have been in place since fiscal year 2013 to ensure a sufficient budget to offer the number of consultations required for safe childbirth (up to 14 visits). Additionally, the “Desired Standards for Health Examinations for Pregnant Women” issued in 2015 stipulates the timing, frequency, and content of prenatal examinations (Japanese Ministry of Health, Labour and Welfare, 2015). Under these circumstances, whereas publicly funded items in prenatal examinations to maintain and improve the health of pregnant women and children has increased, the outcomes of this policy have not been studied in depth. Our project, Pregnant Women Health Initiative (PWHI), was launched in 2018 to clarify the effects of early-pregnancy screening for infectious diseases on health promotion in pregnant women and children during pregnancy and after delivery. In this study, we focused on hepatitis B virus (HBV), hepatitis C virus (HCV), rubella, syphilis, human T-lymphotropic virus type 1 (HTLV-1), and cervical cancer caused by persistent human papillomavirus infection as infectious diseases to be screened in the early stage of pregnancy because these have major effects on the health of both pregnant women and children.

In this report, as a part of our initiative, we conducted a questionnaire survey on the accuracy of pregnant women’s understanding regarding the results of infectious diseases screened during early pregnancy and knowledge about these diseases. A previous study has shown that knowledge about mother-to-child transmission of infectious diseases among Japanese women varies greatly depending on the disease (Morioka et al., 2014); however, there have been no studies on the background and factors associated with pregnant women’s understanding of the test results and knowledge about infectious diseases. In this study, we endeavored to understand the level of pregnant women’s health awareness regarding infectious diseases and clarify the specific roles of medical staff, including obstetricians, midwifes, and local governments, in promoting the health of women and children during pregnancy and after delivery.

## Methods

As part of our project, we recruited pregnant women who were willing to participate in the study between May 2018 and September 2019, with the cooperation of the departments of obstetrics in eight university hospitals and 17 regional core hospitals with perinatal wards across Japan. A written explanation of the study objective and a document containing a study participant number and a quick response (QR) code were distributed to pregnant women who had a delivery appointment at any of the cooperating institutions. Those who wished to participate in the study submitted a signed written consent form including the participant number to their physician and then accessed the study secretariat using the QR code to complete enrollment. The participants then received an email from the study secretariat containing a URL to access the secure online questionnaire, which they completed after entering their assigned participant number. The questionnaire survey was conducted using the online questionnaire tool “Survey Monkey” with enhanced security via Secure Sockets Layer encryption. Participants completed the first questionnaire during their pregnancy, and the gestational age was not specified. The survey included questions about their background, test results, and knowledge of the diseases of interest in this study. We also used data from the medical records of the included pregnant women, which were provided by the cooperating physicians for analysis of the survey responses. In this study, among the examined diseases, we first compared test results reported on the questionnaire and those collected from the medical records to determine the rates of agreement in the results of screening tests for hepatitis B surface antigen (HBsAg), HCV antibody, syphilis screening, HTLV-1 antibody, and cervical cytology as positive for atypical squamous cells of undetermined significance (ASC-US) and worse. On this questionnaire, there was no information on whether the women were treated for these infectious diseases. Next, we investigated whether participants had knowledge of the following four infectious diseases that are transmissible from mother to infant and what factors were associated with their knowledge according to their response to the following question: “Among HBV, HCV, syphilis, and HTLV-1, which are directly relevant to children’s health?” Multiple responses were allowed; the correct answer is “all four diseases.” Note that rubella was excluded from the analysis because the results of a survey on this disease have already been published (Iwata et al., 2020). Additionally, knowledge regarding cervical cytology was excluded from the analysis regarding the effect on the child because this may differ among individuals if high-grade lesions or invasive cervical cancers were found. Because the average household income in Japan is approximately 5.5 million JPY in 2018 (Japanese Ministry of Health, Labour and Welfare, 2019), we defined household income < 5 million JPY as low income, 5–7 million JPY as middle income, and > 7 million as high income.

### Statistical Analysis

Agreement rates between early-pregnancy screening results in the medical records and questionnaire responses for the five items (HBV, HCV, syphilis, HTLV-1, and cervical cytology) were determined using the Kappa coefficient. Logistic regression analysis was used to detect the differences in participants’ knowledge of HBV, HCV, syphilis, and HTLV-1, according to their clinical characteristics and socioeconomic background, such as age, parity, and educational and economic levels. Odds ratios (ORs) and 95% confidence intervals (95% CIs) were used to determine whether differences remained after adjustment for each background variable. The statistical significance level was *p* < 0.05. IBM SPSS version 25 was used for statistical analysis (IBM Corp., Armonk, NY, USA). Non-respondents to the required questions were excluded from the analysis.

## Results

**Fig. 1:**
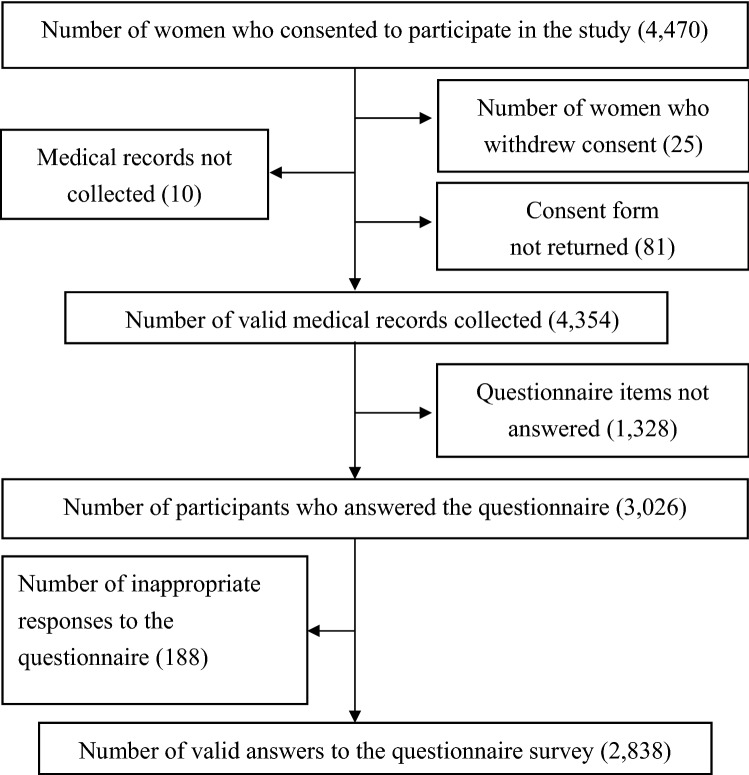
Flowchart of study participants

Of the 4,470 pregnant women who consented to participate in the study, we excluded those with any errors on the consent form or missing data in the medical records of interest in this study; medical records for the remaining 4,354 women were available. Of the 4,354 study participants, 1,328 women did not complete the questionnaire, and 188 were excluded because of inappropriate questionnaire responses. Finally, 2,838 respondents were included in the analysis of this study (Fig. 1). The background variables of questionnaire respondents are summarized in Table 1. Most participants were in their 30 s (66.6%), and the proportions of primiparous and multiparous women were 50.2% and 49.7%, respectively.

**Table 1:** Background characteristics of pregnant Japanese women who completed the questionnaire in 25 centers between 2018 and 2019

Characteristics (*N* = 2,838)	*N*	%
Age at enrollment, y		
20–29	632	22.3
30–39	1,889	66.6
40–49	317	11.2
Number of previous births		
0	1,427	50.2
≥ 1	1,411	49.7
Educational level		
Junior high school and high school graduate	538	19.0
Junior college graduate	961	33.9
College/university graduate	1,339	47.2
Smoking before pregnancy		
No	2,449	86.3
Yes	389	13.7
Annual household income, JPY		
< 5 million	921	32.5
5–7 million	827	29.1
> 7 million	1,090	38.4
Positive results in screening for infectious diseases and abnormal cervical cytology results		
HBV	15	0.53
HCV	3	0.11
Syphilis	9	0.32
HTLV-1	10	0.35
Cervical cytology	72	2.54

*HBV *hepatitis B virus, *HCV* hepatitis C virus, *HTLV*-1 human T-lymphotropic virus type 1

Table 2 shows the positive rates for the five screening test items, obtained from 2,838 medical records, and questionnaire responses regarding screening test results reported by study participants who had abnormal results. Among the first screening results for the five tests, abnormal results were most common for cervical cytology (72 women, 2.54%), followed by HBV (15 women, 0.53%). Additionally, the number of questionnaire responses from women who had normal results but answered “abnormal” is shown. The agreement rates for HBV screening and cervical cytology results were “substantial,” with Kappa coefficients of 0.749 and 0.623, respectively. The rate of agreement for syphilis was “moderate” with a Kappa coefficient of 0.452, and that for HCV and HTLV-1 was “fair” with Kappa coefficients, respectively.

**Table 2:** Positive rates in screening of four infectious diseases and abnormal cervical cytology results, obtained from medical records among women who completed the questionnaire, their answers to the questionnaire of their results, and number of questionnaire answer who tested normal but answered “abnormal”

	Women with positive screening test results or abnormal in cervical cytology, n	Questionnaire answer of women who tested positive in screening or abnormal in cervical cytology, n: Abnormal	Questionnaire answer of women who tested positive in screening or abnormal in cervical cytology, n: Normal	Questionnaire answer of women who tested normal, n: Abnormal	Κappa coefficient
HBV	15	12	3	5	0.749
HCV	3	1	2	3	0.363
Syphilis	9	5	4	7	0.452
HTLV-1	10	4	6	6	0.398
Cervical cytology	72	43	29	18	0.623

HBV, hepatitis B virus; HCV, hepatitis C virus; HTLV-1, human T-lymphotropic virus type 1

Kappa coefficient: < 0 No agreement, 0–0.20 Slight, 0.21–0.40 Fair, 0.41–0.60 Moderate, 0.61–0.80 Substantial, 0.81–1.0 Almost perfect

Agreement rates between screening results from medical records and questionnaire responses for five items (HBV, HCV, syphilis, HTLV-1, cervical cytology) were determined using the Kappa coefficient (*N* = 2,838)

**Fig. 2:**
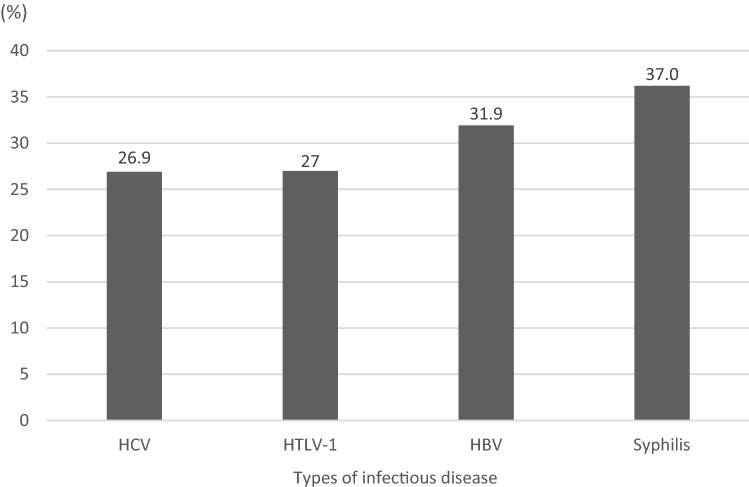
Rates of understanding regarding infectious diseases transmitted from pregnant women to infants HBV, hepatitis B virus; HCV, hepatitis C virus; HTLV-1, human T-lymphotropic virus type 1

Figure 2 depicts the percentages of respondents answering correctly to questions intended to determine whether they knew that the infectious diseases of interest could affect their children. The correct response rate for syphilis was the highest (37.0%); this rate for the other diseases was around 30%. Tables 3 and 4 show the background factors related to correct response rates for HBV, HCV, syphilis, and HTLV-1, respectively. Knowledge about HBV was greater in multiparous women than in primiparous women (adjusted OR 1.141, *p* = 0.015) and higher with higher education levels: junior college graduates (adjusted OR 1.533, *p* = 0.001) and college or university graduates (adjusted OR 1.355, *p* = 0.020) had greater knowledge than high school graduates. Knowledge increased with annual income: groups with incomes > 7 million JPY (adjusted OR 2.145, *p* < 0.001) and 5–7 million JPY (adjusted OR 1.459, *p* < 0.001) had higher knowledge levels than the group with income < 5 million JPY. Knowledge about HCV was greater among junior college graduates than high school graduates (adjusted OR 1.447, *p* = 0.007) and higher among those with a higher annual incomes: the groups with income > 7 million JPY (adjusted OR 2.270, *p* < 0.001) and 5–7 million JPY (adjusted OR 1.633, *p* < 0.001) had greater HCV knowledge than the group with income < 5 million JPY.

**Table 3:** Multivariate analysis of knowledge among pregnant women regarding whether HBV and HCV affect children, stratified by socioeconomic variables, using data from the Pregnant Women Health Initiative, 2018

Characteristics (*N* = 2,838)	Knowledge about HBV		Adjusted OR^a^ (95% CI)	p value	Knowledge about HCV		Adjusted OR^a^ (95% CI)	p value
	Know (%)	Don’t know (%)			Know (%)	Don’t know (%)		
Age group								
20–29	187 (29.6)	445 (70.4)	1.00		147 (23.3)	485 (76.7)	1.00	
30–39	606 (32.1)	1,283 (67.9)	0.889 (0.722–1.094)	0.265	515 (27.3)	1,374 (72.7)	1.013 (0.811–1.264)	0.811
40–49	111 (35.0)	206 (65.0)	0.989 (0.735–1.332)	0.943	102 (32.2)	215 (67.8)	1.242 (0.912–1.691)	0.912
Number of previous birth								
0	434 (30.4)	993 (69.6)	1.00		388 (27.1)	1,039 (72.8)	1.00	
≥ 1	470 (33.3)	941 (66.7)	1.141 (1.026–0.270)	0.015	376 (26.6)	1,035 (73.4)	1.034 (0.924–1.158)	0.561
Educational level								
≤ High school graduate	123 (21.9)	415 (77.1)	1.00		103 (19.1)	435 (80.9)	1.00	
Junior college graduate	323 (33.6)	638 (66.4)	1.533 (1.192–1.970)	0.001	274 (28.5)	687 (71.5)	1.447 (1.109–1.888)	0.007
College/university graduate	458 (34.2)	881 (65.8)	1.355 (1.049–1.750)	0.020	387 (28.9)	952 (71.1)	1.246 (0.950–1.634)	0.112
Smoking before pregnancy								
No	799 (32.6)	1,650 (67.4)	1.00		674 (27.5)	1,775 (72.5)	1.00	
Yes	105 (27.0)	284 (73.0)	0.832 (0.723–1.200)	0.583	90 (23.1)	299 (76.9)	0.963 (0.738–1.256)	0.779
Household income (in ten thousands of yen)								
< 500	212 (23.0)	709 (77.0)	1.00		166 (18.0)	755 (82.0)	1.00	
500–700	257 (31.1)	570 (68.9)	1.459 (1.173–1.814)	0.001	224 (27.1)	603 (72.9)	1.633 (1.294–2.080)	< 0.001
> 700	435 (39.9)	655 (60.1)	2.145 (1.735–2.652)	< 0.001	374 (34.3)	716 (65.7)	2.270 (1.810–2.848)	< 0.001

^a^Model includes all variables for which values are shown in the column

*HBV* hepatitis B virus, *HCV* hepatitis C virus, *OR* odds ratio, *CI* confidence interval

**Table 4:** Multivariate analysis of knowledge among pregnant women regarding whether syphilis and HTLV-1 affect children, stratified by socioeconomic variables, using data from the Pregnant Women Health Initiative, 2018

Characteristics (*N* = 2,838)	Knowledge about syphilis		Adjusted OR^a^ (95% CI)	p value	Knowledge about HTLV-1		Adjusted OR^a^ (95% CI)	p value
	Know (%)	Don’t know (%)			Know (%)	Don’t know (%)		
Age group								
20–29	198 (31.3)	434 (68.7)	1.00		149 (23.6)	483 (76.4)	1.00	
30–39	712 (37.7)	1,177 (62.3)	1.037 (0.845–1.271)	0.729	525 (27.8)	1,364 (72.2)	1.024 (0.821–1.277)	0.836
40–49	124 (39.1)	193 (60.9)	1.069 (0.798–1.431)	0.654	93 (29.3)	224 (70.7)	1.076 (0.786–1.473)	0.647
Number of previous birth								
0	504 (35.3)	923 (64.7)	1.00		377 (26.4)	1,050 (73.6)	1.00	
≥ 1	530 (37.6)	881 (62.4)	1.070 (0.963–1.189)	0.207	390 (27.6)	1,021 (72.4)	1.043 (0.932–1.169)	0.462
Educational level								
≤ High school graduate	126 (23.4)	412 (76.6)	1.00		95 (17.7)	443 (82.3)	1.00	
Junior college graduate	355 (36.9)	606 (63.1)	1.623 (1.268–2.078)	< 0.001	258 (26.8)	703 (73.2)	1.532 (1.166–2.012)	0.002
College/university graduate	553 (41.3)	786 (58.7)	1.628 (1.268–2.089)	< 0.001	414 (30.9)	925 (69.1)	1.620 (1.230–2.134)	0.001
Smoking before pregnancy								
No	937 (38.3)	1,512 (61.7)	1.00		678 (27.7)	1,771 (72.3)	1.00	
Yes	97 (24.9)	292 (75.1)	0.686 (0.531–0.887)	0.004	89 (22.9)	300 (77.1)	0.998 (0.764–1.304)	0.990
Household income (in ten thousands of yen)								
< 500	244 (26.5)	677 (73.5)	1.00		186 (20.2)	735 (79.8)	1.00	
500–700	288 (34.8)	539 (65.2)	1.350 (1.094–1.666)	0.005	193 (23.3)	634 (76.7)	1.115 (0.883–1.407)	0.362
> 700	502 (46.1)	588 (53.9)	2.026 (1.652–2.484)	< 0.001	388 (35.6)	702 (64.4)	1.923 (1.543–2.395)	< 0.001

*OR* odds ratio, *CI* confidence interval, *HTLV*-1 human T-lymphotropic virus type 1

^a^Model includes all variables for which values are shown in the column

Knowledge about syphilis was greater in women with higher education levels; college or university graduates had greater syphilis knowledge than high school graduates (adjusted OR 1.628, *p* < 0.001). Smokers had lower syphilis knowledge than non-smokers (adjusted OR 0.686, *p* = 0.004), and those with annual income of > 7 million JPY had greater knowledge than those with annual income of < 500 million JPY (adjusted OR 2.026, *p* < 0.001). Knowledge about HTLV-1 increased with education level; college or university graduates had greater knowledge levels than high school graduate (adjusted OR 1.620, *p* = 0.001). HTLV-1 knowledge was also greater among those with higher annual incomes: those with income > 7 million JPY (adjusted OR 1.923, *p* < 0.001) had greater knowledge levels than those with incomes < 5 million JPY.

## Discussion

Our survey clarified the following two facts: first, among the pregnant women in our study, understanding of infectious disease screening results in early pregnancy was in good agreement with the actual test results in the medical records, indicating that pregnant women perceived their own test results fairly well. Second, the rates of knowing that HBV, HCV, syphilis, and HTLV-1, tested for in early-pregnancy screening, are transmissible to children was very low.

No questionnaire surveys among pregnant women in Japan have been previously reported regarding the understanding of screening results for pathogens with a risk of vertical transmission from mother to child. Although some women had positive results recorded in the first screening during early pregnancy, those who responded that their test results were “negative” on the questionnaire included those whose results were clinically negative for agents with a risk of mother-to-child transmission in a follow-up or second in-depth examination. Conversely, some women had negative results but reported that their results were “positive.” Most of these responders seemed to indicate that they had normal results in the first screening during pregnancy but recognized their results as abnormal because they had a previous history of abnormal cervical cytology or were currently being followed up for cervical dysplasia. However, we were unable to understand the reason for some wrong answers. These discrepancies may have arisen because the questionnaire simply queried the results of the first screening and participants may have misunderstood the questionnaire. Although the number was small, some pregnant women perceived their test results incorrectly. Health care providers need to be aware of the possibility that some women may misunderstand or forget their test results and must provide careful explanations and appropriate follow-up throughout the pregnancy and after delivery. Moreover, pregnant women who tested positive during screening were likely to include both those who were identified as having an infectious disease for the first time during the prenatal examination and those who were identified as having an infection before pregnancy but did not undergo follow-up. The challenge is to guide these women to undergo long-term follow-up to maintain good health status. In our project, the ongoing second survey at 18 months after delivery is expected to reveal the follow-up status of women with positive results, which has never been systematically investigated in Japan.

The role of perinatal medical facilities is to provide detailed explanation regarding the need for maternal care, the risk of mother-to-child disease transmission, and appropriate preventive measures for pregnant women with positive results in early-pregnancy screening or follow-up testing. Additionally, it is important to facilitate seamless follow-up, such as referring women and their infants to facilities offering postpartum health management; however, there are limitations in ensuring reliable follow-up under patient self-management. The data health revolution to extend healthy life expectancy, which started in Japan in 2020 (Japanese Ministry of Health, Labour and Welfare 2020), may help in solving this problem. The Ministry of Health, Labour, and Welfare has requested medical institutions to provide local governments with the results of prenatal examinations, including test results for infectious diseases during pregnancy (Japanese Ministry of Health, Labour and Welfare 2020). With this initiative, the results of prenatal examinations will be made available to all local governments in the near future. However, at present, the results are only recorded and available in keeping with the regulations of each local government. This reform is expected to help local governments to provide seamless, long-term maternal health management after prenatal examinations. Local governments can use the results as fundamental data to design improved measures against infectious diseases, child abuse prevention, and to provide precise health guidance according to the current health condition of pregnant women, including adequate care after delivery. Also in 2020, the same year, public subsidization was begun to help meet the costs of follow-up examination for those who tested positive in hepatitis screening during prenatal examinations, and local governments introduced information services for eligible residents (Kanagawa Prefectural Government, 2021). In Japan, routine HBV vaccination only began in 2016; the participants in this study were not subject to routine vaccination, and universal HBV vaccination for newborns has not been implemented. Furthermore, a previous study reported that internal medicine consultations for pregnant women with HBV are not sufficiently recommended (Kawasaki et al., 2016). Maternity facilities should provide women who test positive in screening with information on these new services to facilitate in-depth examinations and early treatment.

Because mother-to-child HTLV-1 transmission occurs primarily via breastfeeding (Moriuchi et al., 2013), it is important for health care providers to explain transmission via breastfeeding to pregnant women who are HTLV-1 carriers and guide them to make their own choices about how to feed their children. In Japan, short-term breastfeeding for < 90 days and the use of frozen–thawed breast milk has been recommended as prevention measures, apart from than the exclusive use of artificial milk. The reason is that the infection rate did not differ between the artificial nutrition-only group and the group with short-term breastfeeding, within 90 days of birth. Considering the advantages of breastfeeding (Takezaki et al., 1997), and the frozen breastfeeding method, in which infected T lymphocytes in breast milk are inactivated by freezing, these recommendations prevent mother-to-child infection (Ando et al., 2004). However, the guidelines concerning breastfeeding among women with HTLV-1 were revised in 2017 to recommend exclusive formula feeding, given the insufficient scientific evidence for the effectiveness of previously recommended methods (Itabashi, 2017). Maternity facilities are expected to play a role in the follow-up of women with HTLV-1 infection in terms of managing breast problems associated with the termination of breastfeeding in addition to the provision of up-to-date information.

Regarding cervical cancer screening via Pap smear, the positive rate for ASC-US or worse was 2.9%, higher than the positive rates for the other items. In Japan, the uptake of Pap tests every 2 years, as stated in the Japanese guideline (Japanese Ministry of Health, Labour and Welfare 2004), among the target group of women 20 years and older is very low at around 40% (Sauvaget et al., 2016). Pap tests in early pregnancy are included in routine prenatal testing. However, endocervical sample collection is difficult for cervical cytology tests during pregnancy and rarely yields appropriate cells. The status of participation in cervical cancer screening after childbirth should be clarified using questionnaire surveys after delivery, and not during pregnancy, to increase awareness about proper participation in screening. The problem of Pap tests during pregnancy was also found in a case report of vaginal transmission of cervical cancer from women with advanced cervical cancer to their infants; some women in that study had negative Pap smear results during pregnancy (Arakawa et al., 2021). There have only been a few case reports of transplacental or vaginal transmission of cervical cancer from mother to child. Although this is considered very rare, it should be noted that such transmission can lead to serious consequences for the child (Arakawa et al., 2021; Herskovic et al., 2014).

As the second important finding in this study, only approximately 30% of pregnant women had correct knowledge regarding mother-to-child transmissible diseases tested in early-pregnancy infectious disease screening (Fig. 2). A single-center study among Japanese women reported that at least 60% of women were aware of mother-to-child transmission of HBV, syphilis, and HCV, but only approximately 20% were aware of vertical transmission of HTLV-1 (Morioka et al., 2014). The rates in our study regarding correct understanding of diseases that are transmissible from mother to child were lower than the rates in the previous study, presumably because ours was a multicenter study and responses were collected from a larger number of women. The slightly higher rate of awareness about syphilis than the rates for other infectious diseases may be attributable to increased opportunities to learn about syphilis because of an increasing trend in the number of patients with syphilis in Japan since 2012 (Japanese Ministry of Health, Labour and Welfare, 2021). However, reports of heterosexual syphilis transmission have been increasing since 2014 (National Institute of Infectious Diseases (Japan) 2020); in parallel, the number of reported cases of congenital syphilis has shown an increasing trend (National Institute of Infectious Diseases (Japan) 2021). A previous study showed that 27.8% of mothers in Tanzania knew that syphilis can be transmitted to their children during pregnancy (Chotta et al., 2020). Another study in Bangladesh showed that 13% of women had knowledge about syphilis and there was association about awareness of syphilis with age, residential location, educational level, and socioeconomic status (Hossain et al., 2017). These studies indicate that improving knowledge about syphilis is important in all countries, including Japan.

Thus, even understanding of syphilis is inadequate, as is that of other diseases. Regarding HBV, the number of people infected via blood transfusion has decreased in Japan because the screening of blood for transfusion or production of blood-derived products started in 1972. However, asymptomatic carriers resulting from nonadherence to preventive measures for mother-to-child transmission or the occurrence of prenatal infections and horizontal infections owing to sexual transmission are ongoing problems (National Institute of Infectious Diseases (Japan) 2013). In a 2017 report, the percentages of Japanese women who tested positive for HBsAg, HCV antibodies, syphilis, and HTLV-1 antibodies were 0.3% (Tanaka et al., 2010), 0.3–0.8% (Shiraki, 2005), 0.025% (Suzuki et al., 2017), and 0.16–0.17% (Itabashi, 2013), respectively. We presume that these low prevalence rates are linked to the low awareness of infectious diseases. However, given the increase in horizontal infections via sexual transmission, women should have proper knowledge regarding infectious diseases before they become pregnant. They need to understand how the respective infectious diseases can be transmitted to them, what the imminent infection risks are, and what problems their children might face in the case of mother-to-child transmission. Efforts to increase awareness in this population of women regarding all of these infectious diseases are necessary.

As for the background factors affecting women’s level of understanding, there were no differences in terms of age; parity was also not a factor, except for HBV. These results suggest that women’s knowledge about and preventive actions against infectious diseases do not improve with the experience of childbirth, as predicted in our previous report, displaying no association between parity and a history of rubella vaccination (Iwata et al., 2020). A population with higher social status, such as women with a higher educational background or higher annual income, is likely to have more opportunities to acquire knowledge about infectious diseases. Because it is not possible for pregnant women to prevent contracting infections or mother-to-child transmission only by being careful, a broader segment of the population, including men and non-pregnant women, should be educated about infectious diseases.

### Limitations

The participants in this study were pregnant women who delivered in general or university hospitals with obstetric services mainly for high-risk pregnant women as well as women who were willing to respond to the questionnaire. Therefore, the possibility of selection bias should be taken into account. Moreover, because this was a simple questionnaire survey, the participants’ intended response was unclear for some questions, which may affect the interpretation of the responses.

## Conclusions for Practice

The results of this study indicated that our study population of pregnant women in Japan had generally accurate understanding of their results in early-pregnancy infectious disease screening. However, they had inadequate knowledge regarding infectious diseases transmitted from pregnant women to infants. In Japan, data health reform has recently begun, and information on prenatal examinations is now managed digitally rather than in the form of the Maternal and Child Health Handbook used previously for self-management. These data are expected to soon be used for various purposes, such as health management by local governments and big data analysis. To use the information from prenatal examinations for long-term health management of pregnant women and children, it is crucial to implement effective and organized perinatal checkups and follow-ups for infectious diseases via collaboration between health care providers in perinatal care facilities and those in local governments.

## Data Availability

The datasets used in the analyses of the current study are available from the corresponding author on reasonable request.
